# In-Flight Transmission of SARS-CoV-2

**DOI:** 10.3201/eid2611.203254

**Published:** 2020-11

**Authors:** Edward M. Choi, Daniel K.W. Chu, Peter K.C. Cheng, Dominic N.C. Tsang, Malik Peiris, Daniel G. Bausch, Leo L.M. Poon, Deborah Watson-Jones

**Affiliations:** London School of Hygiene & Tropical Medicine, London, UK (E.M. Choi, D.G. Bausch, D. Watson-Jones);; The University of Hong Kong, Hong Kong, China (D.K.W. Chu, M. Peiris, L.L.M. Poon);; Department of Health, The Government of Hong Kong Special Administrative Region, Hong Kong (P.K.C. Cheng, D.N.C. Tsang);; Public Health England, London (D.G. Bausch);; National Institute for Medical Research, Mwanza, Tanzania (D. Watson-Jones)

**Keywords:** 2019 novel coronavirus disease, coronavirus disease, COVID-19, severe acute respiratory syndrome coronavirus 2, SARS-CoV-2, viruses, respiratory infections, zoonoses, air travel, disease transmission, infectious, aircraft, whole-genome sequencing, genome, viral, case reports, epidemiologic studies

## Abstract

Four persons with severe acute respiratory syndrome coronavirus 2 infection had traveled on the same flight from Boston, Massachusetts, USA, to Hong Kong, China. Their virus genetic sequences are identical, unique, and belong to a clade not previously identified in Hong Kong, which strongly suggests that the virus can be transmitted during air travel.

In 2019, severe acute respiratory syndrome coronavirus 2 (SARS-CoV-2) emerged in China, ultimately causing the coronavirus disease (COVID-19) pandemic. Many persons with SARS-CoV-2 infection have since flown into and out of COVID-19–affected areas (*1*). Some countries quarantine arriving passengers. Airports are also screening passenger body temperatures before boarding and after arrival. Recent investigations have shown that SARS-CoV-2 can be transmitted before symptom onset, posing a challenge to outbreak control (*2*). Although risks for SARS-CoV-2 transmission have been extensively investigated, in-flight transmission of the virus has not been formally confirmed. Airline staff members have voiced concerns over acquisition of SARS-CoV-2 infection (*3*). Given that flights are still departing to and from COVID-19–affected countries, determining whether in-flight transmission of SARS-CoV-2 occurs is essential.

## The Study

We examined public records for 1,110 persons with laboratory-confirmed COVID-19 in Hong Kong, China, recorded from January 23 through June 13, 2020; we used Centre for Health Protection (CHP) public records and the Vote4HK COVID-19 in HK database for case-patients who had traveled before diagnosis (*4*,*5*). At the time, the Hong Kong government had yet to introduce mandatory quarantine and airport screening (*6*). We identified a cluster of 4 persons with COVID-19 (henceforth referred to as patients A–D) associated with a commercial flight that departed from Boston, Massachusetts, USA, on March 9 and arrived in Hong Kong on March 10, 2020. The airplane, a Boeing 777-300ER, flew for »15 hours and carried a maximum of 294 passengers. The cluster comprised 2 passengers and 2 cabin crew members. Although these persons did not fulfill the criteria for SARS-CoV-2 testing at the time of arrival, results of reverse transcription PCR conducted in local healthcare settings within 5–11 days of arrival were positive. All 4 case-patients subsequently recovered (Appendix Figure 1).

Patients A and B were a married couple. Patient A was a 58-year-old man with underlying disease who sat in a window seat in business class on the airplane (Appendix Figure 2). On March 10, fever and productive cough developed; on March 13, he had mild abdominal discomfort, followed by diarrhea 2 days later. His 61-year-old wife, patient B, also had underlying illness. She sat directly in front of him in a business class window seat. On March 10, she had a sore throat. One day later, fever and cough developed. As their symptoms evolved, they sought healthcare and were hospitalized on March 14. On March 15, respiratory samples (collected March 14 for patient A and March 15 for patient B) were positive for SARS-CoV-2. No public record indicates what their underlying diseases were or whether these 2 passengers were symptomatic during the flight. Before the flight and within the 14-day incubation period, they visited Toronto, Ontario, Canada (February 15–March 2); New York, New York, USA (March 2–5); and Boston (March 5–9). CHP classified the couple as imported cases into Hong Kong.

Patient C was an asymptomatic 25-year-old man identified through contact tracing by the Hong Kong government and the airline as a close contact of patients A and B. He was a Hong Kong–based business class flight attendant who served patients A and B during the flight. After patients A and B received their diagnoses, the airline informed patient C, and he attended an outpatient clinic on March 16. He was positive for SARS-CoV-2 on March 17 and was subsequently quarantined and hospitalized. Patient C stayed in Boston during March 5–9. Patient D was a 51-year-old female Hong Kong–based flight attendant on the same flight. Fever and cough developed on March 18, SARS-CoV-2 test result was positive on March 21, and patient D was hospitalized. There is no publicly available information of her travel history before the flight or her contacts with the other patients on or after the flight. Descriptions of the disease experienced by patients C and D were unavailable. CHP categorized patients C and D as close contacts of a person with an imported case.

To generate genetic evidence for transmission between the 4 patients, we sequenced their viruses. Samples were collected under public health authority, and individual patient identities are known to CHP. Retrospective analysis of leftover samples without individual consent was permitted under local regulations and approved by the institutional review board of the University of Hong Kong/Hospital Authority West Cluster (reference UW 20-168) and the London School of Hygiene & Tropical Medicine Ethics Committee (reference 22384). Stored upper respiratory samples were sent to a World Health Organization reference laboratory at the University of Hong Kong. We deduced near full-length genomes (sequence length >29,760 nt) by using the Illumina sequencing method and previously described primers and protocol (*7*). All deduced sequences had a minimum coverage of 100. While sequencing and analyzing the specimens, we were blinded to patient status as passenger or crew.

The near full-length viral genomes from all 4 patients were 100% identical and phylogenetically grouped to clade G (Figure). Other than these 4, none of the 189 viral sequences deduced from samples collected in Hong Kong (January 21–May 12, 2020; GISAID, http://platform.gisaid.org), belong to this clade (data not shown) (K.S. Leung et al., unpub. data, https://www.medrxiv.org/content/10.1101/2020.03.30.20045740v2). Conversely, in March 2020, virus sequences related to those of patients A–D with only 2 nt differences were isolated in Toronto, New York City, and Massachusetts (Table), making it plausible that patients A and B acquired a similar virus during their visit. Worldwide during January 10–June 13, »30,000 complete SARS-CoV-2 genomes with high coverage were deposited into the GISAID database. None shares 100% identity with the sequences of the viruses in the cluster reported here.

**Figure:**
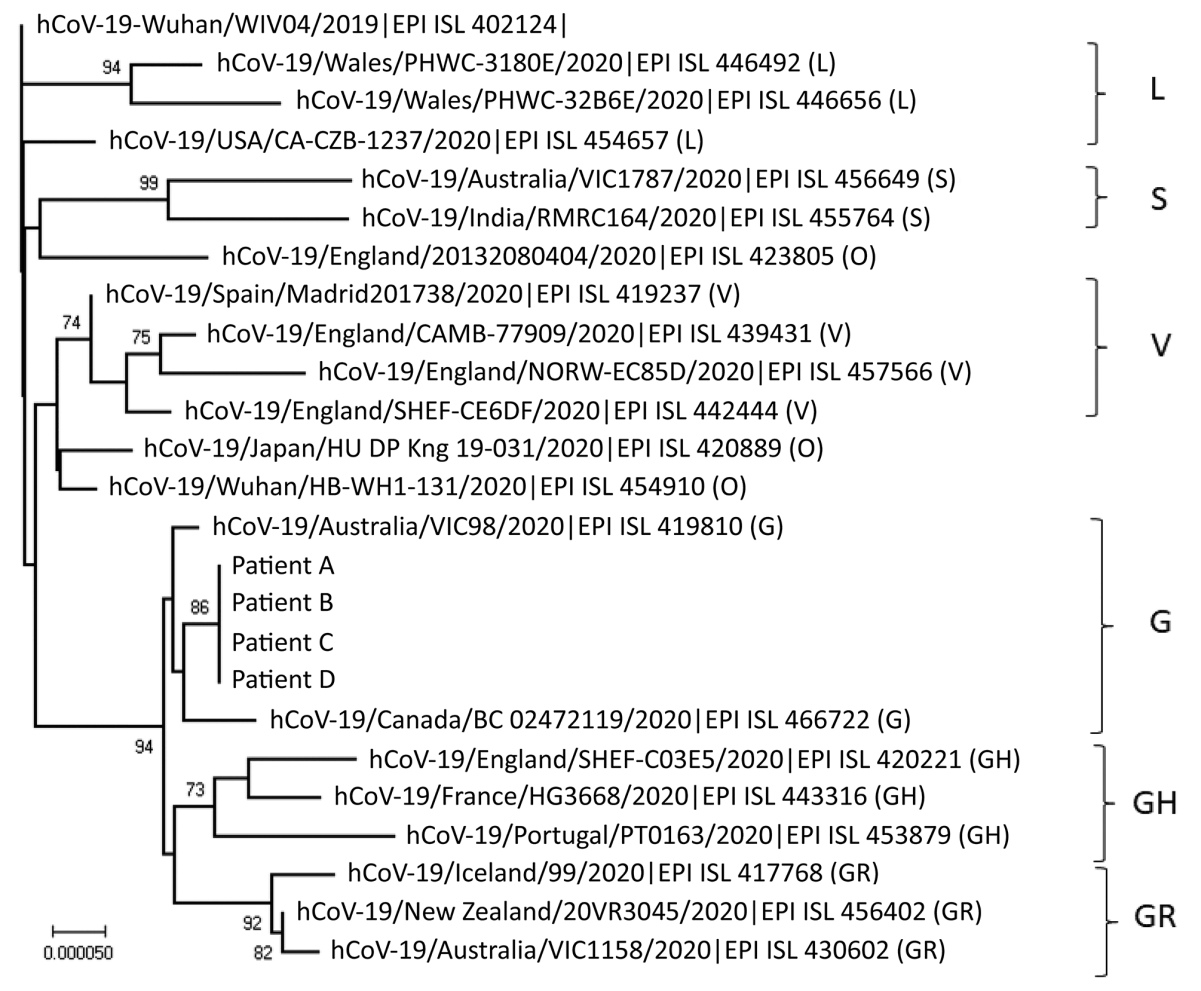
Phylogenetic tree of the severe acute respiratory syndrome coronavirus 2 (SARS-CoV-2) viruses isolated from passengers and airline crew members who traveled on the same flight from Boston, Massachusetts, USA, to Hong Kong, China. Human SARS-CoV-2 WIV04 is selected to be the root of this phylogenetic tree. The tree was constructed by using the neighbor-joining method. Only bootstrap values >80 are shown. Representative viruses from clades L, S, V, G, GH, GR, and O (others) are included in the analysis. Virus sequences from patients A–D reported in this study are grouped to clade G (GISAID [http://platform.gisaid.org] accession nos. EPI_ISL_476801 to EPI_ISL_476804). EPI ISL accession nos. for sequences retrieved in GISAID (http://platform.gisaid.org) are provided. Scale bar indicates estimated genetic distance.

**Table:** Single nucleotide polymorphisms in the SARS-CoV-2 virus sequences from 4 patients on the same flight from Boston, Massachusetts, USA, to Hong Kong, China*

GISAID accession no.	Source	Sample collection date	Nucleotide positions†						Nucleotide difference‡
			241	3037	9857	11083	14408	23403	
402124	Reference sequence WIV04†	2019 Dec 30	C	C	C	G	C	A	6
476802	Patient A	2020 Mar 14	T	T	T	T§	T¶	G#	0
476801	Patient B	2020 Mar 15	T	T	T	T§	T¶	G#	0
476803	Patient C	2020 Mar 17	T	T	T	T§	T¶	G#	0
476804	Patient D	2020 Mar 19	T	T	T	T§	T¶	G#	0
460471	Massachusetts, USA	2020 Mar 27	T	T	C	G	T¶	G#	2
427528	New York, USA	2020 Mar 12	T	T	C	G	T¶	G#	2
418354	Ontario, Canada	2020 Mar 15	T	T	C	G	T¶	G#	2

*These 4 viral genomes are unique among the sequences deposited into the GISAID database (http://platform.gisaid.org) January 10–June 13, 2020. They contain 6 polymorphisms compared with the WIV04 reference sequence of SARS-CoV-2, 3 of which are nonconservative. SARS-CoV-2 genomes that differ from these by 2 nt had been reported from Massachusetts, USA; New York, USA; and Ontario, Canada, in March 2020. SARS-CoV-2, severe acute respiratory syndrome coronavirus 2. †A 2019 reference sequence from a patient in Wuhan, China (hCoV-19/Wuhan/WIV04/2019). The nucleotide positions shown are relative to this reference sequence (GISAID accession ID EPL_ISL_402124). ‡No. nucleotide differences relative to the virus genomes of patients A–D. §Nonconservative polymorphism at nucleotide position 11083, which corresponds to a Leu (TTG) to Phe (TTT), L37F, amino acid change in the NSP6 protein. ¶Nonconservative polymorphism at nucleotide position 14408, which corresponds to a Pro (CCT) to Leu (CTT), P323L, amino acid change in the NSP12 protein. #Nonconservative polymorphism at nucleotide position 23403, which corresponds to an Asp (GAT) to Gly (GGT), D614G, amino acid change in the spike protein.

## Conclusions

Given the case histories and sequencing results, the most likely sequence of events is that one or both of passengers A and B contracted SARS-CoV-2 in North America and transmitted the virus to flight attendants C and D during the flight. The only location where all 4 persons were in close proximity for an extended period was inside the airplane. Passengers and cabin crew do not generally go through the same check-in process at airports before boarding. Although we cannot completely rule out the possibility that patients C and D were infected before boarding, the unique virus sequence and 100% identity across the whole virus genome from the 4 patients makes this scenario highly unlikely. Patient D may have acquired infection from patient C, but because their test results were positive within 1 incubation period, it is more likely that patient D was infected by patient A or B. We therefore conclude that these 4 patients belong to the same in-flight transmission chain.

Our results strongly suggest in-flight transmission of SARS-CoV-2. No other COVID-19 cases associated with this flight have been identified. We were unable to quantify the virus attack rate on this flight because not all passengers were tested.

Previous reports of probable in-flight transmissions of SARS-CoV-2 lack genetic evidence (*8*,*9*). During January–March 2020, the International Air Transport Association received 3 reports of suspected in-flight transmission (*10*). Contact tracing of 2 passengers who flew from China to Canada has yielded no indication of secondary infections from the flight (*11*). Nonetheless, SARS-CoV-2 test results have been positive for hundreds of flight attendants and pilots; at least 2 have died (*12*,*13*). Our results demonstrate that SARS-CoV-2 can be transmitted on airplanes. To prevent transmission of the virus during travel, infection control measures must continue.

Appendix Additional methods and results for study of in-flight transmission of severe acute respiratory syndrome coronavirus 2.
