# Supplementary material for: In-Flight Transmission of SARS-CoV-2
Source: Emerg Infect Dis. 2020 Nov;26(11):2713–6. doi: 10.3201/eid2611.203254 (PMC7588512; doi:10.3201/eid2611.203254)
Supplement: Appendix — Additional methods and results for study of in-flight transmission of severe acute respiratory syndrome coronavirus 2. [file 20-3254-Techapp-s1.pdf]

Article DOI: https://doi.org/10.3201/eid2611.203254

In-Flight Transmission of Severe Acute Respiratory Syndrome Coronavirus 2

Appendix

Methods

Database search and case identification

In this study, we used publicly available data on COVID-19 cases in Hong Kong, including government records compiled by the Centre for Health Protection (CHP), Department of Health in Hong Kong, the Vote4HK "COVID-19 in HK" public database and newspaper reports. The CHP regularly releases reports of COVID-19 cases diagnosed in Hong Kong. These CHP public reports do not have personal identifiers and contain information on the cases' symptoms, date of onset, date of diagnosis, number of close contacts, the presence of pre-existing conditions (yes/no) and travel history, including inbound travelers' seats on aircrafts. The Vote4HK "COVID-19 in HK" database collates data on the CHP records and provides links to newspaper reports on government briefings of COVID-19 cases. We examined 1110 cases in the Hong Kong between 23 January and 13 June 2020, and identified passengers and cabin crew who had previously travelled in the same aircraft.

Specimen collection and laboratory analysis

This paper describes four RT-PCR-confirmed COVID-19 cases on a flight that landed in Hong Kong on 10 March 2020. Since 19 February 2020, the Hospital Authority and the CHP have been running the Enhanced Laboratory Surveillance Programme and offered free testing for SARS-CoV-2 to patients with fever and respiratory symptoms or mild chest infection, especially those who travelled outside Hong Kong within 14 days.(*1*) From 8 March 2020, all inbound travelers arriving at the Hong Kong International Airport have to complete health declaration form (*2*). Respiratory samples from persons meeting the case definition are tested for SARS-CoV-2 using RT-PCR at the Public Health Laboratory Services, a WHO reference laboratory at

CHP (*3*). These include nasopharyngeal aspirates, nasopharyngeal swabs, throat swabs and saliva.

The four cases in our transmission cluster did not fulfil the criteria for SARS-CoV-2 testing upon arrival, but were tested in local healthcare settings within 5-11 days. Stored samples of these cases were then sent to a WHO reference laboratory at the University of Hong Kong for full genome analyses. Near full-length genomes (N≥ 29760 nucleotides) were deduced by Illumina sequencing method using the primers and protocol previously described by us (*4*). All the deduced sequences had a minimum coverage of 100 or above. The specimens were sequenced and analyzed blind to the passenger/crew/case status of the four individuals.

Representative sequences from each phylogenetic clade of SARS-CoV-2 (G, GH, GR, L, O, S and V) were retrieved from GISAID. Viral sequences were aligned and phylogenetically analyzed using BioEdit and MEGA-X, respectively. A phylogenetic tree was constructed by the neighbor-joining method with bootstrap testing (N=1,000). Metadata from 191 Hong Kong viral sequences deposited in GISAID were also used in the analyses.

Data sharing

The virus genome sequencing results can have been deposited into the GISAID database (http://platform.gisaid.org). The accession numbers are EPI_ISL_476801 to EPI_ISL_476804.

Bias and missing data

In the absence of mandatory SARS-CoV-2 screening for all the passengers and crew members from the 9 March 2020 Boston to Hong Kong flight, only symptomatic COVID-19 cases and known contacts of those and other cases were identified, leading to a potential underestimation of the true number of cases who acquired the infection on board. Hong Kong has an active surveillance system in place to track and trace every COVID-19 case within its territory. However, it is possible that individuals with asymptomatic or mildly symptomatic infection are missing from the public databases.

References

1. The Government of Hong Kong SAR. HA enhanced laboratory surveillance programme extends to outpatients with mild symptoms [cited 2020 Jun 24]. https://www.info.gov.hk/gia/general/202002/18/P2020021800437.htm

2. The Government of Hong Kong SAR. DH strengthens health declaration measure at Hong Kong International Airport and urges the public to delay non-essential travel outside Hong Kong [cited 2020 Jun 24]. https://www.info.gov.hk/gia/general/202003/06/P2020030600821.htm

3. Poon L, Chu D, Peiris M. Detection of 2019 novel coronavirus (2019-nCoV) in suspected human cases by RT-PCR [cited 2020 Jun 24]. https://www.who.int/docs/default-source/coronaviruse/whoinhouseassays.pdf

4. Sit THC, Brackman CJ, Ip SM, Tam KWS, Law PYT, To EMW, et al. Infection of dogs with SARS-CoV-2. Nature. 2020 May 14. Epub ahead of print. PubMed https://doi.org/10.1038/s41586-020-2334-5

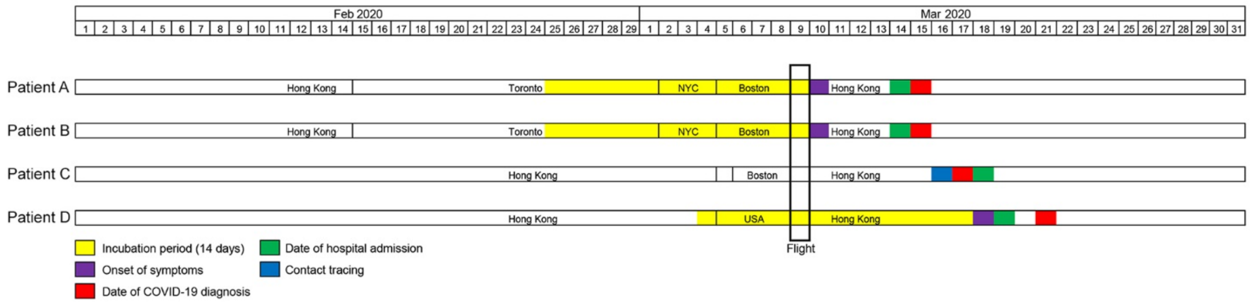

Appendix Figure 1. Timeline of events surrounding in-flight transmission of SARS-CoV-2 to 4 passengers who traveled on the same airplane from Boston, Massachusetts, USA, arriving in Hong Kong, China, on March 10, 2020. Because patient C was asymptomatic at the time of diagnosis, no incubation period has been indicated.

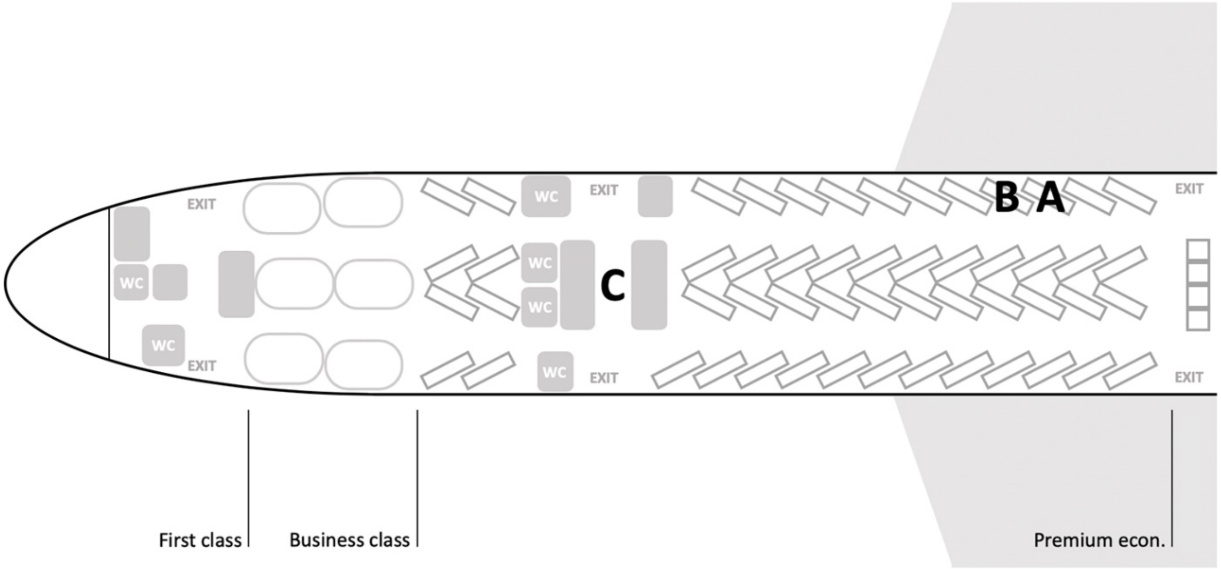

Appendix Figure 2. Seating plan and locations of COVID-19 patients on the Boeing 777-300ER aircraft during the Boston to Hong Kong flight. Index patients A and B were business class passengers sitting in adjacent rows in individual window-facing seats. Patient C was a business class flight attendant who served patients A and B during the flight. Patient D was working as a flight attendant on the same flight, but there is no public information on the aircraft sections that the attendant worked in. This diagram is based on information from the airline’s website. Econ., economy.
